# Basic Reproduction Number of Chikungunya Virus Transmitted by *Aedes* Mosquitoes

**DOI:** 10.3201/eid2610.190957

**Published:** 2020-10

**Authors:** Najmul Haider, Francesco Vairo, Giuseppe Ippolito, Alimuddin Zumla, Richard A. Kock

**Affiliations:** The Royal Veterinary College, University of London, London, UK (N. Haider, R.A. Kock);; National Institute for Infectious Diseases Lazzaro Spallanzani, Rome, Italy (F. Vairo, G. Ippolito);; University College London, London (A. Zumla)

**Keywords:** Basic reproduction number, R_0_, chikungunya virus, outbreaks, epidemic, *Aedes* mosquito, vector-borne infections, mosquito-borne infections, viruses, arboviruses, zoonoses

## Abstract

We estimated the weighted mean basic reproduction number (R_0_) of chikungunya virus based on outbreak size. R_0_ was 3.4 (95% CI 2.4–4.2) and varied for 2 primary chikungunya mosquito vectors: 4.1 (95% CI 1.5–6.6) for *Aedes aegypti* and 2.8 (95% CI 1.8–3.8) for *Ae. albopictus*.

The basic reproduction number (R_0_) of an infection is the mean number of secondary cases a single infectious person causes in a completely susceptible population. The magnitude of R_0_ is used to measure the risk and spread of an epidemic or pandemic. To control an outbreak, the R_0_ should be reduced to <1 through interventions, such as vaccination. Because little information is available at the beginning of an epidemic, the estimated R_0_ commonly is used to assess public health preparedness needs, the impact of the possible epidemic, and success of the control measures. Information on R_0_ often is lacking for emerging diseases like chikungunya, a mosquito-borne viral disease of humans and nonhuman primates.

Chikungunya virus (CHIKV) is a member of the Alphavirus genus (family Togaviridae) transmitted by *Aedes* mosquitoes, primarily *Ae. aegypti* and *Ae. albopictus*. *Ae. aegypti* mosquitoes are aggressive human biters and the main vectors for CHIKV outbreaks in Asia, where epidemics occur primarily in urban settings (*1*). *Ae. albopictus* mosquitoes, on the other hand, feed from several mammals besides humans and are responsible for CHIKV outbreaks in rural and urban areas in Africa (*1*).

CHIKV outbreaks were reported from >100 countries worldwide during 2014–2019 (*2*). Epidemiologic understanding of CHIKV changed after outbreaks on the island of La Réunion in the Indian Ocean during 2005–2006, when *Ae. albopictus* mosquitoes were identified as the outbreak vector (*1*,*3*). The global expansion of CHIKV partially is attributed to viral adaptation to this new mosquito vector, which facilitated a mutation in the coding for the envelop protein 1 A226V (E1-A226V) gene of CHIKV, increasing the competence of *Ae. albopictus* mosquitoes to transmit the virus from mosquitoes to humans (*1*–*3*).

In humans, CHIV infection is characterized by sudden onset of intense polyarthralgia, high fever, and skin rash. CHIKV causes debilitating joint pain that can limit daily activities and last a few months to several years (*2*); progression to the chronic stage (>3 months) occurs in 4.1%–78.6% of cases (*4*). To estimate R_0_ of CHIKV outbreaks, we analyzed empirical data on R_0_ available from open sources.

## The Study

We used the search terms “Basic reproduction number” or “R_0_” AND “chikungunya” to identify published articles from Google Scholar and PubMed. We identified 11 articles describing estimated R_0_ of CHIKV from outbreak data during 2000–2019. We found 5 articles on outbreaks in Africa, all on La Réunion (*3*,*5*–*8*); 1 on an outbreak in Cambodia (*1*); 2 on outbreaks in Italy (*9*,*10*); and 3 on outbreaks in the Americas (*11*,*12*; N. Báez-Hernández et al. unpub. data, https://www.biorxiv.org/content/10.1101/122556v1).

The authors estimated R_0_ by using mathematical (compartmental) models fitted with respective outbreak data (*1*,*3*,*5*–*12*). We considered the estimated values comparable and extracted the R_0_ from each. We then estimated the weighted mean R_0_ of CHIKV based on outbreak size, such as number of reported cases included in the estimation of R_0_ in the original article, and further estimated the mean R_0_ for different mosquito vectors and E1-A226V gene mutations.

The largest CHIKV outbreak occurred on La Réunion and affected 266,000 of the 785,000 inhabitants (*3*). Several models with differing levels of data estimated the R_0_ of the La Réunion outbreaks between 0.89 and 4.1 (*3*,*5*–*7*). The R_0_ also was estimated from CHIKV outbreaks in Italy in 2007 (*10*) and 2017 (*9*), Cambodia in 2012 (*1*), Venezuela in 2014 (*11*), Colombia in 2015 (*12*), and Mexico in 2015 (N. Báez-Hernández et al. unpub. data, https://www.biorxiv.org/content/10.1101/122556v1) (Table).

**Table:** The basic reproduction number (R_0_) of chikungunya virus estimated from empirical outbreak data, 2000–2019

Year	Country or region	Continent	R_0_ range (95% CI)	Mosquito species	Lineage	E1-A226V mutation*	Reference
2006	La Réunion	Africa	4.1	*Ae. albopictus*	Indian Ocean	Y	(*3*)
2006	La Réunion	Africa	0.9–2.3	*Ae. albopictus*	Indian Ocean	Y	(*7*)
2006	La Réunion	Africa	1.5–1.8	*Ae. albopictus*	Indian Ocean	Y	(*5*)
2006	La Réunion	Africa	3.4	*Ae. albopictus*	Indian Ocean	Y	(*6*)
2006	La Réunion	Africa	3.7 (2–11)	*Ae. albopictus*	Indian Ocean	Y	(*8*)
2007	Italy	Europe	3.3 (1.8–6.0)	*Ae. albopictus*	Indian Ocean	Mixed	(*10*)
2012	Cambodia	Asia	6.5 (6.2–6.8)	*Ae. aegypti*	Asian	Y	(*1*)
2014	Italy	Europe	2.1 (1.5–2.6)	*Ae. albopictus*	Indian Ocean	N	(*9*)
2014	Venezuela	South America	3.7	*Ae. aegypti*	Asian	N	(*11*)
2015	Mexico	North America	3.44	*Ae. aegypti*	Asian	N	†
2014	Colombia	South America	1–9	*Ae. aegypti*	Asian	N	(*12*)
*Envelope 1 A226V gene. †N. Báez-Hernández et al., unpub data, https://www.biorxiv.org/content/10.1101/122556v1.							

We estimated the weighted mean R_0_ of CHIKV to be 3.4 (95% CI 2.4–4.2). We analyzed the data and estimated the R_0_ for *Ae. aegypti* and *Ae. albopictus* mosquitoes separately for outbreaks in which the R_0_ of CHIKV was described for each species. We estimated the R_0_ to be 4.1 (95% CI 1.5–6.6) for *Ae. aegypti* mosquitoes and 2.8 (95% CI 1.8–3.8) for *Ae. albopictus* mosquitoes. Although the difference is not statistically significant (p = 0.12), we expected a lower R_0_ for outbreaks involving *Ae. albopictus* mosquitoes because this species also feeds on animals, which might have reduced the attack rate on humans and transmission across the population. However, outbreaks associated with *Ae. albopictus* mosquitoes can be prolonged and the outbreak response can have economic consequences. We estimated the R_0_ to be 3.5 (95% CI 1.9–4.9) during outbreaks involving the E1-A226V mutation, which is higher than R_0_ of 2.1 from the 2017 outbreak in Italy that did not have the gene mutation.

CHIKV infections among humans can have severe health consequences, despite the low case fatality rate. CHIKV infection has 3 stages: acute, postacute, and chronic. The acute phase usually lasts for 1–3 weeks and is characterized by fever, intense myalgia, arthralgia, and symmetric joint pain in both legs that can limit even the simplest daily activities. The postacute stage usually lasts 1–3 months after the acute phase and is characterized by persistent inflammatory arthralgia, arthritis, tenosynovitis, and bursitis. The chronic stage starts after 3 months and can last for months to years after acute infection (*2*).

In a study in Brazil, >68% of persons with CHIKV remained chronically infected for up to 1 year (*13*). On Réunion Island, a small group of patients had clinical signs for 6 years. Although the reason for persistence is unclear, it might be strain related and associated with the E1-A226V mutation. Therefore, despite being less severe and causing fewer deaths than other mosquitoborne diseases, CHIKV can have lingering physical and psychological consequences for those affected. Infected persons also can experience economic consequences because they might not be able to work for several weeks or more.

R_0_ does not remain constant. For arboviruses, R_0_ can vary based on the density of hosts and vectors; mosquito species, survival, and biting rate; and vector competence and capacity, all of which can depend on environmental and microclimatic factors. Further, the vector competence of *Ae. aegypti* mosquitoes for CHIKV might be different from that for *Ae. albopictus* mosquitoes, which could influence outbreak dynamics. For example, 1 study reported the transmission efficiency of *Ae. albopictus* mosquitoes as 97% and of *Ae. aegypti* mosquitoes as 83% (*14*). 

The outbreaks included in our study occurred in tropical and subtropical countries and in the more temperate climate of Italy. We did not consider climatic conditions during reported outbreaks, which might play a role in determining the size and R_0_ of CHIKV outbreaks. We also did not consider the variation of data quality in published articles, except for the outbreak size, which might affect estimated R_0_. However, defining adjustments for data quality would have been difficult and might have introduced unwanted bias.

## Conclusions

We found the overall mean R_0_ for CHIKV was 3.4 (95% CI 2.4–4.2). Our estimated R_0_ of 4.1 (95% CI 1.5–6.6) for *Ae. aegypti* mosquitoes suggests CHIKV could spread rapidly and cause high disease incidence in urban areas, where this species thrives. Our estimated CHIKV R_0_ for *Ae. albopictus* mosquitoes of 2.8 (95% CI 1.5–6.6) was lower than for *Ae. aegypti* mosquitoes. In rural areas, where *Ae. albopictus* mosquitoes are more prevalent, sylvatic cycles, maintenance of biodiversity including natural mosquito populations, and presence of hosts other than humans might reduce the effects of an outbreak. Early interventions targeting *Aedes* mosquitoes will be vital to controlling CHIKV outbreaks.
